# Observations of playground play during elementary school recess

**DOI:** 10.1186/s13104-018-3861-0

**Published:** 2018-10-23

**Authors:** William V. Massey, Byungmo Ku, Megan B. Stellino

**Affiliations:** 1 College of Public Health and Human Sciences, School of Biological and Population Health Sciences, Kinesiology Program, Oregon State University, 101 Milam Hall 2520 SW Campus Way, Corvallis, OR 97331 USA; 2 College of Public Health and Human Sciences, School of Biological and Population Health Sciences, Kinesiology Program, Oregon State University, 008 Women’s Building, Corvallis, OR 97331 USA; 3 School of Sport and Exercise Science, University of Northern Colorado, Greeley, CO 80639 USA

**Keywords:** Play, Recess, Physical activity, School-based health, Reliability, Validity

## Abstract

**Objective:**

The purpose of the current study was to examine reliability and validity evidence for an observational measure of playground play during recess. Observational data of what children played at recess were collected at 236 recess sessions across 26 urban elementary schools. An inductive content analysis of children’s type of play and activity engagement during recess was conducted to categorize activities. Inter-rater reliability of observations was assessed at 49 points that spanned 22 unique recess periods at four different schools. Reliability data were collected during the winter and spring seasons. A multivariate analysis of variance was conducted to examine differences in play and activity patterns between genders, and between schools implementing recess interventions (e.g., structured play environment) and schools with no recess intervention.

**Results:**

Results of the content analysis yielded eight playground play and activity categories, all with high levels of inter-rater reliability (ICCs > .90). Significant differences in children’s play and activity patterns emerged between genders and across recess intervention conditions. Engagement in ‘sports and organized activities’ and ‘non-engagement in play’ contributed most to the separation between boys and girls, while ‘non-engagement in play’ contributed most to the separation between recess intervention and non-intervention schools.

## Introduction

School-based recess is an opportunity for children to be physically active during the school day [1]. The American Academy of Pediatrics [2] has asserted that in the United States school-based recess not only provides an environment for health enhancing physical activity (PA), but also confers benefits for social development, cognitive functioning, and improved classroom behavior. Yet, results of observational research show that high levels of fighting and conflict can occur during recess, particularly in schools serving high levels of socially disadvantaged students [3, 4]. There is a need, therefore, to consider the quality of the recess environment, which has recently been operationalized as including the safety and structure of the environment, adult engagement and supervision, student social behaviors, and transition periods [5].

Inherent in developing a quality recess environment is understanding children’s preferences and opportunities to engage in, and play different games and activities. While previous research has shown differences between boys and girls PA levels at recess [1], and that environmental interventions are effective for increasing PA at recess [6], less attention has been given to children’s play preferences that may contribute to both PA promotion, as well as social development during recess. The activities of daily living-playground play (ADL-PP) is an existing measure that was developed for self-reported activities in children older than 7 years of age [7, 8]. Stellino and Sinclair [9] found that children (N = 444; 3rd–5th graders; 49% female) from a suburban Rocky Mountain region of the USA reported running (79.5%) and talking with friends (60.8%) as the most common activities at recess. However, self-report of activities can be prone to selection or retrieval biases, and Sinclair and Stellino [10] further suggested that this tool may be adaptable to an observational form of measurement. While previous research has shown that observers can be trained to enhance reliability on the ADL-PP, and that agreement between observer reports and children self-report is generally around 75% [7], these data were produced in small recess sessions ranging from eight to 29 participants. In considering the observational use of the ADL-PP, researchers may be able to examine larger samples of what children do at recess, optimal levels of activity engagement, and better tailor interventions to the needs and general play preferences of children (e.g., more targeted approach to buying equipment and structuring environments). Based on these premises, we used a modified version of the ADL-PP to assess the reliability and validity of an observational measure of children’s play patterns.

## Main text

Data in the current study were collected in Milwaukee, WI, USA from 2014 to 2017. A waiver of consent was approved by the institutional review board at Concordia University Wisconsin (ID: 932380-3; 926512-1; 594622-5) in line with category 1 (standard educational procedures) and category 2 (observations of public behavior) exempted research categories and US Code of Federal Regulations 46.117.

### Participants

Data were collected at 236 different recess sessions across 26 elementary schools. On average, 65 children were present at each recess session, resulting in approximately 16,150 individual observations.

### Measures

The different types of activities children engaged in during recess were measured using an observational form of the ADL-PP [9]. Observations were conducted at 5-min intervals during recess, with frequency counts being collected for all different games, activities, or sedentary behaviors. Throughout data collection, a second coder recorded inter-rater reliability observations at 49 points that spanned 22 unique recess periods at four different schools. To facilitate independent reliability ratings, data assessors were instructed not to discuss frequency counts with other raters, however coordinated their data collection to ensure they conducted their observations at the same time and in the same direction (i.e., counts all took place left to right to control for natural momentary changes in play). Observations for boys and girls were coded separately. Over the course of observations, 80 different activities were recorded including those within the original ADL-PP and those marked in the ‘other’ category.

Aside from the ADL-PP, observers recorded whether or not an intervention was present on the playground. In total, 107 of the observed recess sessions had a recess intervention present on the playground (n = 82 recess sessions contracted by Playworks, a national non-profit organization; *n *=25 recess sessions running internal interventions designed by trained physical education teachers).

### Data analysis

Following data collection, an inductive content analysis of activity engagement was conducted by the lead author [11]. Activities that were redundant were combined into a singular category (e.g., straight slide, tube slide, curly slide, were all coded into slide). Next categories of activities were coded into higher order groupings (e.g., slide was coded into jungle gym). These were then sent to the third author for critical peer review with any discrepancies being solved through discussion. Finally, the literature on children’s play at recess was reviewed to ensure we included activities known to take place at recess but not observed in this study (e.g., use of cell phones or electronics). The results of this content analysis were the basis for creating an observational protocol for playground play derived from use of the original ADL-PP (Table 1). Next, inter-rater reliability of observational coding for each play domain was tested using a one-way random effects intra-class correlation coefficient (ICC).

**Table 1:** “Observation of Playground Play” (OPP) tool for use in coding children’s behavior

Playing on equipment (frequency count)		
Jungle gym	Boys	Girls
Swings	Boys	Girls
Rock climbing	Boys	Girls
Other	Boys	Girls
Organized sports and activities (frequency count)		
Basketball	Boys	Girls
Football	Boys	Girls
Kickball	Boys	Girls
Soccer	Boys	Girls
Dance	Boys	Girls
Other	Boys	Girls
Active and chase games (frequency count)		
Tag/chasing	Boys	Girls
Racing	Boys	Girls
Running	Boys	Girls
Other	Boys	Girls
Traditional playground games (frequency count)		
Four square	Boys	Girls
Hula-hoops	Boys	Girls
Jump rope	Boys	Girls
Tetherball	Boys	Girls
Hopscotch	Boys	Girls
Unfixed equipment	Boys	Girls
Other	Boys	Girls
Nature (frequency count)		
Imaginative play	Boys	Girls
Digging	Boys	Girls
Climbing hills	Boys	Girls
Playing with bugs	Boys	Girls
Other	Boys	Girls
Rough and tumble play (frequency count)		
Wrestle	Boys	Girls
Play fight	Boys	Girls
Fence climbing	Boys	Girls
Other	Boys	Girls
Anti-social behavior (frequency count)		
Fighting	Boys	Girls
Pushing/shoving/hitting	Boys	Girls
Timeout/wall	Boys	Girls
Yelling	Boys	Girls
Other	Boys	Girls
Non-engagement in active play (frequency count)		
Standing around	Boys	Girls
Talking with friends	Boys	Girls
Electronics	Boys	Girls
Sit-down game	Boys	Girls
Other	Boys	Girls

To test the construct validity of the tool, differences between gender, and presence of intervention, were examined using a factorial (2 × 2) multivariate analysis of variance (MANOVA) test in SAS 9.4 (©SAS Institute, Inc., North Carolina, USA). Based on previous research, it was hypothesized that different patterns would emerge between boys and girls, and between children at intervention schools and non-intervention schools [1, 6]. Canonical coefficients were also computed to determine the relative contribution (i.e., largest, smallest) of each dependent variable to the identified discriminant function. An alpha of .05 was used to determine statistical significance for all omnibus tests, and reported effect sizes (η^2^) were interpreted as small (η^2^ = .01), medium (η^2^ = .06), or large (η^2^ = .14). Post-hoc univariate tests were then conducted. The relative benefit increase of the presence of an intervention for boys and girls separately as it relates to engagement during recess (Engagement Rate at Intervention Schools—Engagement Rate at Non-Intervention Schools/Engagement Rate at Non-Intervention Schools) was also examined.

Statistical assumptions were checked prior to conducting the omnibus tests. Three categories (anti-social behaviors; rough and tumble play; nature) violated normality assumptions, which was likely based on low prevalence rates. Nonetheless, we viewed these categories as having practical significance in considering children’s recess play activities. Given previous reports that ANOVA based analyses are robust to violations of normality [12, 13] in conjunction with issues surrounding log transformations of frequency data [14, 15], the decision was made to not transform the data to meet normality assumptions, as this could compromise meaningful interpretation of the data.

### Results

Content analyses of the activities children engaged in during recess yielded eight categories of play: (1) playing on equipment, (2) organized sports and activities, (3) active and chasing games, (4) traditional playground games, (5) nature, (6), rough and tumble play, (7) anti-social behavior, and (8) non-engaged in play. Table 1 depicts a new observational form derived from the ADL-PP, the *Observation of Playground Play (OPP).* Inter-rater reliability coefficients for each category are reported in Table 2.

**Table 2:** Descriptive statistics and reliability indices

OPP play domain	Total	Boys	Girls	Intervention	Non-intervention	Inter-rater reliability
Playing on equipment	11.38%	10.51%	12.25%	14.15%^++^	9.09%	*ICC *= .986
Organized sports and activities	26.54%	41.60%^+^	11.48%	26.61%	26.48%	*ICC *= .965
Active and chase games	14.51%	12.91%^+^	16.11%	15.14%	13.98%	*ICC *= .969
Traditional playground games	15.74%	14.10%^+^	17.39%	20.72%^++^	11.61%	*ICC *= .939
Nature	1.38%	1.14%	1.63%	1.70%	1.12%	*ICC *=.999
Rough and tumble play	1.27%	1.00%	1.61%	1.07%	1.43%	*ICC *= .972
Anti-social behavior	1.72%	1.78%	1.67%	1.17%^++^	2.18%	*ICC *= .999
Non-engagement in active play	27.49%	17.06%^+^	37.92%	19.40%^++^	34.20%	*ICC *= .976

^+^Significant difference between boys and girls

^++^Significant differences between intervention and non-intervention

Descriptive statistics for children’s playground play engagement patterns were calculated and are reported in Table 2. The factorial MANOVA test result revealed a non-significant interaction effect, *F*(8, 461) = 1.85, *p *= .07, Λ = .97, η^2^ = .03). A significant main effect of gender was identified, F(8, 461) = 41.32, p < .001, Λ = .58, η^2^ = .42. The standardized canonical coefficients reported in Table 3 revealed that in the context of all eight play categories, organized sports and activities, and non-engagement contributed most to the separation between males and females. Post hoc tests revealed that boys engaged in significantly lower levels of non-engagement t(470) = 13.198, p < .001, traditional playground games t(470) = 2.426, p = .016, active and chase games t(470) = 2.559, p = .011, and higher levels of organized sports and activities t(470) = − 17.898, p < .001, when compared to girls. A significant main effect for intervention was also identified, F(8, 461) = 5.26, p < .001, Λ = .916, η^2^ = .08. The standardized canonical coefficients revealed that non-engagement contributed most to the separation between schools with and without an intervention. Post hoc tests revealed that children at intervention schools engaged in higher levels of playing on equipment t(470) = − 3.789, p < .001 and traditional playground games t(470) = − 6.991, p < .001, and lover levels of non-engagement t(470) = 8.555, p < .001 and anti-social behavior t(421.53) = 2.152, p = .032. Finally, at schools with an intervention present, there was a relative benefit increase of 45.8% to reduce non-engagement for boys and a relative benefit increase of 42% to reduce non-engagement for girls.

**Table 3:** Canonical coefficients associated with gender and intervention contrasts

OPP play domain	Gender standardized canonical coefficient	Intervention standardized canonical coefficient
Playing on equipment	− .126	.709
Organized sports and activities	.626	1.264
Active and chase games	− .094	.562
Traditional playground games	− .294	.536
Nature	− .125	.440
Rough and tumble play	− .138	.591
Anti-social behavior	.200	.274
Non-engagement in active play	− .903	2.076

### Discussion

The current study extends the school-based (PA) measurement literature. Evidence is provided for an observational measure of children’s playground play across eight activity categories [16]. The *Observations of Playground Play (OPP)* measure provides a practical tool for researchers, and practitioners, to better understand playground activity and play patterns in an outdoor environment. In examining playground play patterns, the data in the current study help to elucidate how differential play patterns might explain discrepancies in recess PA, and how interventions might play a role in reducing this gap. Specifically, while PA has been shown to increase physical health, as well as cognitive performance [17], data directly linking recess PA to social, cognitive, or academic outcomes remains elusive [18]. Thus, the type of activity, and the social-emotional implications of engagement (e.g., fighting versus participation in a team sport) are likely to moderate various outcomes, and the use of the *OPP* can help researchers and practitioners better understand how play preferences shape the recess environment.

In terms of validity, the activity patterns in the current study are consistent with previous studies, indicating that gender significantly impacts children’s patterns of activity engagement during recess [1, 19, 20]. In the current study, boys were significantly more involved in organized sports and activities (e.g., soccer and football) and participated less in non-engagement activity (e.g., talking with friends and sit-down games) compared to girls. This finding supports results from previous research [9], that indicated boys participated in more sport-related activities and girls were more engaged in non-active play (e.g., talking), or use of equipment (e.g., swinging). The difference in activity between boys and girls found in the present study is also consistent with other previous findings that examined the meaning of recess for boys and girls. Notably, a study found that boys consider recess as a time for participating in sports-related activity but girls consider it as a time for participating in sedentary-related social activity [21].

Examining differences between intervention and non-intervention schools also supported the construct validity of the OPP. Compared to children at recesses with no intervention present, children in recesses with an intervention engaged in playing on equipment significantly more, and showed patterns of non-engagement in active play and anti-social behaviors significantly less. This is in line with previous research that showed recess interventions including, but not limited to adding equipment, play markings, zones, and teacher engagement, are effective for promoting PA [16]. Furthermore, and aligned with previous research, the standardized canonical coefficients reported in the current study revealed that non-engagement in active play contributed most to the separation between schools with, and without, an intervention.

## Limitations

An important limitation of the current study is that schools represented one geographic region of the United States, and the patterns of recess play and activity observed in the current study might not be representative of a broader and/or more national, or international, population. Additional limitations include a lack of concurrent validity of recess play and PA patterns and a lack of detail specifying how the environment shaped various those patterns. Finally, due to the time sampling nature of frequency counts as a mode of observation, it is likely that more sporadic behaviors (e.g., pushing and shoving) are under-represented.
